# COVID-19 Incidence and Death Rates Among Unvaccinated and Fully Vaccinated Adults with and Without Booster Doses During Periods of Delta and Omicron Variant Emergence — 25 U.S. Jurisdictions, April 4–December 25, 2021

**DOI:** 10.15585/mmwr.mm7104e2

**Published:** 2022-01-28

**Authors:** Amelia G. Johnson, Avnika B. Amin, Akilah R. Ali, Brooke Hoots, Betsy L. Cadwell, Shivani Arora, Tigran Avoundjian, Abiola O. Awofeso, Jason Barnes, Nagla S. Bayoumi, Katherine Busen, Carolyn Chang, Mike Cima, Molly Crockett, Alicia Cronquist, Sherri Davidson, Elizabeth Davis, Janelle Delgadillo, Vajeera Dorabawila, Cherie Drenzek, Leah Eisenstein, Hannah E. Fast, Ashley Gent, Julie Hand, Dina Hoefer, Corinne Holtzman, Amanda Jara, Amanda Jones, Ishrat Kamal-Ahmed, Sarah Kangas, FNU Kanishka, Ramandeep Kaur, Saadiah Khan, Justice King, Samantha Kirkendall, Anna Klioueva, Anna Kocharian, Frances Y. Kwon, Jacqueline Logan, B. Casey Lyons, Shelby Lyons, Andrea May, Donald McCormick, MSHI, Erica Mendoza, Lauren Milroy, Allison O’Donnell, Melissa Pike, Sargis Pogosjans, Amy Saupe, Jessica Sell, Elizabeth Smith, Daniel M. Sosin, Emma Stanislawski, Molly K. Steele, Meagan Stephenson, Allen Stout, Kyle Strand, Buddhi P. Tilakaratne, Kathryn Turner, Hailey Vest, Sydni Warner, Caleb Wiedeman, Allison Zaldivar, Benjamin J. Silk, Heather M. Scobie

**Affiliations:** ^1^Epidemiology Task Force, COVID-19 Emergency Response Team, CDC; ^2^Rhode Island Department of Health; ^3^Public Health – Seattle & King County, Seattle, Washington; ^4^District of Columbia Department of Health; ^5^Utah Department of Health; ^6^New Jersey Department of Health; ^7^Michigan Department of Health and Human Services; ^8^New York City Department of Health and Mental Hygiene, New York; ^9^Arkansas Department of Health; ^10^Massachusetts Department of Public Health; ^11^Colorado Department of Public Health and Environment; ^12^Alabama Department of Public Health; ^13^New Mexico Department of Health; ^14^New York State Department of Health; ^15^Georgia Department of Public Health; ^16^Florida Department of Health; ^17^Vaccine Task Force, COVID-19 Response Team, CDC; ^18^Louisiana Department of Health; ^19^Minnesota Department of Health; ^20^Center for Surveillance, Epidemiology, and Laboratory Services, CDC; ^21^Nebraska Department of Health and Human Services; ^22^Wisconsin Department of Health Services; ^23^Idaho Department of Health and Welfare; ^24^Texas Department of State Health Services; ^25^Tennessee Department of Health; ^26^Data Analytics and Visualization Task Force, CDD COVID-19 Emergency Response Team; ^27^Kansas Department of Health and Environment; ^28^Indiana Department of Health.

Previous reports of COVID-19 case, hospitalization, and death rates by vaccination status^†^ indicate that vaccine protection against infection, as well as serious COVID-19 illness for some groups, declined with the emergence of the B.1.617.2 (Delta) variant of SARS-CoV-2, the virus that causes COVID-19, and waning of vaccine-induced immunity (*1*–*4*). During August–November 2021, CDC recommended^§^ additional primary COVID-19 vaccine doses among immunocompromised persons and booster doses among persons aged ≥18 years (*5*). The SARS-CoV-2 B.1.1.529 (Omicron) variant emerged in the United States during December 2021 (*6*) and by December 25 accounted for 72% of sequenced lineages (*7*). To assess the impact of full vaccination with additional and booster doses (booster doses),^¶^ case and death rates and incidence rate ratios (IRRs) were estimated among unvaccinated and fully vaccinated adults by receipt of booster doses during pre-Delta (April–May 2021), Delta emergence (June 2021), Delta predominance (July–November 2021), and Omicron emergence (December 2021) periods in the United States. During 2021, averaged weekly, age-standardized case IRRs among unvaccinated persons compared with fully vaccinated persons decreased from 13.9 pre-Delta to 8.7 as Delta emerged, and to 5.1 during the period of Delta predominance. During October–November, unvaccinated persons had 13.9 and 53.2 times the risks for infection and COVID-19–associated death, respectively, compared with fully vaccinated persons who received booster doses, and 4.0 and 12.7 times the risks compared with fully vaccinated persons without booster doses. When the Omicron variant emerged during December 2021, case IRRs decreased to 4.9 for fully vaccinated persons with booster doses and 2.8 for those without booster doses, relative to October–November 2021. The highest impact of booster doses against infection and death compared with full vaccination without booster doses was recorded among persons aged 50–64 and ≥65 years. Eligible persons should stay up to date with COVID-19 vaccinations.

Weekly COVID-19 cases (April 4–December 25, 2021) and associated deaths (April 4–December 4, 2021) by vaccination status, including additional and booster doses starting October 3, were reported from 25 state and local health departments that routinely link case surveillance to vaccination data from immunization registries; 2- and 5-week reporting lag times for cases and deaths, respectively, allowed for more complete reporting, data linkage, and mortality ascertainment. Standardized definitions were used for COVID-19 cases in fully vaccinated or unvaccinated persons, COVID-19 cases in fully vaccinated persons with booster doses, and COVID-19–associated deaths,** with specimen collection dates used as time points. Partially vaccinated persons were excluded; reinfections occurring after >90 days were counted as new cases, per current guidance. Analysis periods were determined based on variant proportion estimates in the United States.^††^ Age-specific vaccine administration data were used for incidence rate denominators; numbers of unvaccinated persons were estimated by subtracting the numbers of fully and partially vaccinated persons from 2019 U.S. intercensal population estimates.^§§^ A continuity correction assumed at ≥5% of each age group and jurisdiction would always be unvaccinated (i.e., fully vaccinated coverage ≤95%). Average weekly incidences were calculated by age group (18–49, 50–64, and ≥65 years), vaccination status, and primary series vaccine product (Ad.26.COV2.S [Janssen {Johnson & Johnson}], BNT162b2 [Pfizer-BioNTech], and mRNA-1273 [Moderna]) during each period; rates overall and by vaccine product were age-standardized using the 2000 U.S. Census standard population.^¶¶^ IRRs were calculated by dividing incidence among unvaccinated persons by incidence among fully vaccinated persons (overall and by receipt of booster doses); after detrending the underlying linear changes in incidence, 95% CIs were calculated based on the remaining variation in observed weekly rates (*8*,*9*). To interpret IRR changes, age-standardized crude vaccine effectiveness (VE) was estimated as (1 − [incidence in vaccinated / incidence in unvaccinated]). SAS (version 9.4; SAS Institute) and R (version 4.1.0; R Foundation) were used to conduct all analyses. This activity was conducted consistent with applicable federal law and CDC policy.***

During April 4–December 25, 2021, a total of 6,812,040 COVID-19 cases among unvaccinated persons and 2,866,517 cases among fully vaccinated persons were reported among persons aged ≥18 years in 25 U.S. jurisdictions; 94,640 and 22,567 COVID-19–associated deaths among unvaccinated and fully vaccinated persons, respectively, were reported by December 4 (Table 1). Average weekly, age-standardized rates of cases and deaths (events per 100,000 population) were higher during periods of Delta predominance and Omicron emergence than during pre-Delta and Delta emergence periods and were consistently higher in all periods among unvaccinated persons (range = 64.0–725.6 [cases] and 1.5–11.4 [deaths]) than among fully vaccinated persons (range = 7.4–230.9 and 0.1–0.7).

**TABLE 1:** Average weekly age-standardized incidence of COVID-19 cases (April 4–December 25, 2021) and associated deaths (April 4–December 4, 2021) and incidence rate ratios* for unvaccinated and fully vaccinated persons,^†^ by period^§^ — 25 U.S. jurisdictions,^¶^ April–December 2021

Event/Variant emergence/ Predominance period^§^	Unvaccinated persons		Fully vaccinated persons		Average weekly IRR (95% CI)**
	Total no.	Average weekly incidence*	Total no.	Average weekly incidence*	
**COVID-19 cases**					
Pre-Delta (April–May 2021)	1,006,686	163.8	48,111	11.8	13.9 (12.4–15.5)
Delta emergence (June 2021)	196,988	64.0	30,317	7.4	8.7 (6.1–12.4)
Delta predominance (July–November 2021)	4,546,682	460.1	1,862,090	90.9	5.1 (4.3–6.0)
Omicron emergence (December 2021)	1,061,684	725.6	925,999	230.9	3.1 (1.7–5.8)
**Total**	**6,812,040**	**—**	**2,866,517**	—	—
**COVID-19–associated deaths**					
Pre-Delta (April–May 2021)	11,047	2.7	1,016	0.1	21.9 (17.8–26.8)
Delta emergence (June 2021)	3,107	1.5	556	0.1	16.4 (13.2–20.4)
Delta predominance (July–November 2021)	78,256	11.4	20,313	0.7	16.3 (13.8–19.3)
Omicron emergence (first week in December 2021)^††^	2,230	9.7	682	0.5	NC
**Total**	**94,640**	—	**22,567**	—	—

**Abbreviations**: FDA = Food and Drug Administration; IRR = incidence rate ratio; NC = not calculated for the single reported week of deaths in December 2021.

* Events per 100,000 population. Average weekly incidences and rate ratios are provided by age group, primary series vaccine type, and overall; overall and vaccine-specific rates were standardized by age, according to the enumerated 2000 U.S. Census age distribution. IRRs were calculated by dividing incidence among unvaccinated persons by incidence among fully vaccinated persons overall.

^†^ A COVID-19 case in a fully vaccinated person occurred when SARS-CoV-2 RNA or antigen was detected in a respiratory specimen collected ≥14 days after completing the primary series of a COVID-19 vaccine with FDA approval or emergency use authorization. A COVID-19 case in an unvaccinated person occurred when the person did not receive any FDA-authorized COVID-19 vaccine doses before the specimen collection date. Excluded were partially vaccinated persons who had received at least one FDA-authorized or approved vaccine dose but did not complete a primary series ≥14 days before collection of a specimen with SARS-CoV-2 RNA or antigen detected. This analysis represents the combined impact of BNT162b2 (Pfizer-BioNTech), mRNA-1273 (Moderna), and Ad.26.COV2.S (Janssen [Johnson & Johnson]) COVID-19 vaccines, which had different clinical efficacies against confirmed infection; fully vaccinated persons also include those persons who received additional primary doses and booster doses starting in mid-August. A COVID-19–associated death occurred in a person with a documented COVID-19 diagnosis who died, and whose report local health authorities reviewed (e.g., using vital records, public health investigation, or other data sources) to make that determination. Per national guidance, this should include persons whose death certificate lists COVID-19 disease or SARS-CoV-2 as an underlying cause of death or as a significant condition contributing to death. Rates of COVID-19 deaths by vaccination status are reported based on when the patient was tested for COVID-19, not the date they died.

^§^ Four analysis periods were designated based on the threshold week when the weighted proportions of lineages from whole-genome sequencing results submitted to or performed by CDC: pre-Delta (April 4–May 29, 2021: 0.1%–7% proportion range), Delta emergence (May 30–July 3, 2021: 14%–69%), Delta predominance (July 4–November 27, 2021: 81%–99%), and Omicron emergence (November 28–December 25, 2021: 1%–72%).

^¶^ Alabama, Arkansas, California, Colorado, District of Columbia, Florida, Georgia, Idaho, Indiana, Kansas, Louisiana, Massachusetts, Michigan, Minnesota, Nebraska, New Jersey, New Mexico, New York, New York City (New York), Rhode Island, Seattle/King County (Washington), Tennessee, Texas, Utah, and Wisconsin.

** 95% CIs were calculated after detrending underlying linear changes in weekly incidence rates using piecewise linear regression. Each CI represents the remaining variation in observed weekly incidence rates and resulting incidence rate ratios. The number of observations informing each CI reflects the number of weeks per period: April–May (7), June (5), July–November (21), and December (4).

^††^ For deaths, a single week (November 28–December 4, 2021) of data was reported during the December 2021 period of Omicron emergence, but the proportion of Omicron variant during that first week was approximately 1%.

The age-standardized IRR for cases in unvaccinated versus fully vaccinated persons was 13.9 during April–May and progressively declined to 8.7 during June, 5.1 during July–November, and 3.1 during December, coinciding with the periods of Delta emergence, Delta predominance, and Omicron emergence, respectively. This decline suggests a change in crude VE for infection from 93% during April–May, to 89% during June, 80% during July–November, and to 68% during December. Age-standardized IRRs for deaths among unvaccinated versus fully vaccinated persons were relatively stable; crude VE for deaths was 95% during April–May, 94% during June, and 94% during July–November.

Rates of COVID-19 cases were lowest among fully vaccinated persons with a booster dose, compared with fully vaccinated persons without a booster dose, and much lower than rates among unvaccinated persons during October–November (25.0, 87.7, and 347.8 per 100,000 population, respectively) and December 2021 (148.6, 254.8, and 725.6 per 100,000 population, respectively) (Table 2). Similar trends were noted for differences in the mortality rates among these three groups (0.1, 0.6, and 7.8 per 100,000 population, respectively) during October–November. Age-standardized case IRRs among unvaccinated persons compared with fully vaccinated persons with a booster dose declined from 13.9 during October–November to 4.9 during December, representing potential decreases in crude VE for infection from 93% to 80%, respectively. Comparing unvaccinated persons with fully vaccinated persons without a booster dose, age-standardized case IRRs during October–November and December were 4.0 and 2.8 respectively, representing decreases in VE from 75% to 64%. During October–November, age-standardized IRRs for deaths among unvaccinated persons were 53.2 compared with those in fully vaccinated persons with a booster dose and 12.7 compared with persons without a booster dose; these results represented crude VE against death of 98% and 92%, respectively. Protection improved among persons who received a booster dose compared with not receiving a booster, regardless of primary series vaccine product type. Booster doses provided the largest gains in protection among persons aged ≥65 years followed by persons aged 50–64 years when compared with those aged 18–49 years.

**TABLE 2:** Average weekly incidence* of cases and deaths and incidence rate ratios^†^ for unvaccinated compared with fully vaccinated persons^§^ with and without booster doses,^¶^ by age, vaccine type,** and period^††^ — 25 U.S. jurisdictions**^§§^** October 3–December 25, 2021

Event/Time/Characteristic	COVID-19 vaccination status							
	Unvaccinated		Fully vaccinated (no booster dose)			Fully vaccinated (with booster dose)		
	Total no.	Average weekly incidence*	Total no.	Average weekly incidence*	Average weekly IRR (95% CI)^¶¶^	Total no.	Average weekly incidence*	Average weekly IRR (95% CI)^¶¶^
**COVID-19 cases**								
**October–November**								
Overall (age-standardized)	1,108,298	347.8	650,820	87.7	4.0 (3.6–4.4)	19,954	25.0	13.9 (12.2–15.9)
**Age group, yrs**								
18–49	760,042	330.3	343,602	89.9	3.6 (3.2–4.3)	6,265	27.4	12.0 (10.0–14.5)
50–64	225,290	355.3	174,071	86.5	4.1 (3.5–4.8)	4,911	23.2	15.3 (12.8–18.3)
≥65	122,966	403.6	133,147	80.7	5.0 (4.4–5.6)	8,778	18.1	22.3 (19.0–26.1)
**Vaccine product**								
Moderna	NR	NR	219,623	75.0	4.6 (4.2–5.1)	4,911	20.0	17.4 (14.5–21.1)
Pfizer-BioNTech	NR	NR	358,933	93.9	3.7 (3.4–4.1)	14,292	27.1	12.9 (11.4–14.5)
Janssen (Johnson & Johnson)	NR	NR	71,897	107.5	3.2 (2.9–3.6)	745	26.0	13.4 (10.6–16.9)
**December**								
Overall (age-standardized)	1,061,684	725.6	800,940	254.8	2.8 (1.6–5.2)	125,059	148.6	4.9 (2.7–8.9)
**Age group, yrs**								
18–49	781,969	745.6	547,733	302.5	2.5 (1.1–5.6)	65,710	191.7	3.9 (1.8–8.6)
50–64	189,789	680.8	176,639	208.8	3.3 (1.7–6.4)	31,753	97.0	7.0 (3.0–16.3)
≥65	89,926	704.9	76,568	133.5	5.3 (3.3–8.4)	27,596	50.4	14.0 (6.4–30.6)
**Vaccine**								
Moderna	NR	NR	251,784	221.6	3.3 (1.7–6.1)	39,813	130.4	5.6 (3.1–10.1)
Pfizer-BioNTech	NR	NR	473,115	280.1	2.6 (1.4–4.7)	77,844	162.6	4.5 (2.4–8.3)
Janssen (Johnson & Johnson)	NR	NR	75,903	246.6	2.9 (1.8–4.8)	7,377	132.7	5.5 (3.2–9.4)
**COVID-19–associated deaths**								
**October–November**								
Overall (age-standardized)	16,527	7.8	5,493	0.6	12.7 (11.6–13.8)	285	0.1	53.2 (37.5–75.4)
**Age, yrs**								
18–49	2,094	1.0	124	0.0	27.6 (16.3–46.5)	5	0.0	NC***
50–64	4,427	7.3	659	0.4	21.0 (18.9–23.2)	38	0.2	38.0 (17.1–78.9)
≥65	10,006	33.4	4,710	3.1	11.0 (9.8–12.2)	242	0.5	61.4 (47.8–78.9)
**Vaccine**								
Moderna	NR	NR	2,379	0.5	14.6 (13.0–16.4)	96	0.2	40.1 (19.5–82.5)
Pfizer–BioNTech	NR	NR	2,550	0.7	11.8 (10.8–12.9)	187	0.1	58.7 (36.8–93.9)
Janssen (Johnson & Johnson)	NR	NR	560	1.0	7.9 (6.0–10.3)	2	0.1	NC***

**Abbreviations**: FDA = Food and Drug Administration; IRR = incidence rate ratio; NC = not calculated for categories with counts <20; NR = not relevant for individual vaccine types because the comparison being made is to overall unvaccinated counts and age-adjusted rates.

* Events per 100,000 population.

^†^ Average weekly incidence rates and rate ratios are provided by age group, primary series vaccine type, and overall; overall and vaccine-specific rates were standardized by age, according to the enumerated 2000 U.S. Census age distribution. IRRs were calculated by dividing incidence among unvaccinated persons by incidence among fully vaccinated persons (overall and by receipt of booster doses).

^§^ A COVID-19 case in a fully vaccinated person occurred when SARS-CoV-2 RNA or antigen was detected in a respiratory specimen collected ≥14 days after completing the primary series of a COVID-19 vaccine with FDA approval or emergency use authorization. A COVID-19 case in an unvaccinated person occurred when the person did not receive any FDA-authorized COVID-19 vaccine doses before the specimen collection date. Excluded were partially vaccinated persons who had received at least one FDA-authorized or approved vaccine dose but did not complete a primary series ≥14 days before collection of a specimen with SARS-CoV-2 RNA or antigen detected. This analysis represents the combined impact of BNT162b2 (Pfizer-BioNTech), mRNA-1273 (Moderna), and Ad.26.COV2.S (Janssen [Johnson & Johnson]) COVID-19 vaccines, which had different clinical efficacies against confirmed infection; fully vaccinated persons also include those persons who received additional primary doses and booster doses starting in mid-August. A COVID-19–associated death occurred in a person with a documented COVID-19 diagnosis who died, and whose report local health authorities reviewed (e.g., using vital records, public health investigation, or other data sources) to make that determination. Per national guidance, this should include persons whose death certificate lists COVID-19 disease or SARS-CoV-2 as an underlying cause of death or as a significant condition contributing to death. Rates of COVID-19 deaths by vaccination status are reported based on when the patient was tested for COVID-19, not the date they died.

^¶^ A COVID-19 case in a fully vaccinated person with a booster dose occurred when a person had SARS-CoV-2 RNA or antigen detected on a respiratory specimen collected ≥14 days after receipt of at least one additional or booster dose of any COVID-19 vaccine on or after August 13, 2021. On August 13, 2021, CDC recommended an additional Pfizer-BioNTech or Moderna primary series dose for persons with moderately or severely immunocompromise. On September 24, 2021, CDC recommended a Pfizer-BioNTech booster dose for certain Pfizer-BioNTech primary series recipients, including all adults aged ≥65 years and persons aged ≥18 years in certain populations and high risk occupational and institutional settings. On October 21, 2021, CDC recommended a booster dose for adults aged ≥18 years who received the Janssen vaccine and for Pfizer-BioNTech or Moderna primary series recipients, including all adults aged ≥65 years and persons aged ≥18 years in certain populations and high risk occupational and institutional settings. On November 19, 2021, and November 29, 2021, CDC expanded recommendations for booster doses to include all adults aged ≥18 years.

** Reporting is by primary series vaccine type rather than additional or booster dose vaccine type. The booster dose vaccine type may be different than the primary series vaccine type. For primary mRNA vaccination series, the vaccine product of the second dose was used to determine primary series product type. If the Janssen vaccine was either the first dose or the second dose, the series type was called Janssen. The overall fully vaccinated group includes FDA-approved vaccinations of unknown vaccine type.

^†† ^Analysis periods for this table were designated based on robust reporting of cases among persons with booster doses from October 2021 and the threshold week when the weighted proportions of lineages from whole-genome sequencing results submitted to or performed by CDC: October 3–November 27, 2021 (Delta predominance: 99%), and November 28–December 25, 2021 (Omicron emergence: 1%–72%).

^§§^ Alabama, Arkansas, California, Colorado, District of Columbia, Florida, Georgia, Idaho, Indiana, Kansas, Louisiana, Massachusetts, Michigan, Minnesota, Nebraska, New Jersey, New Mexico, New York, New York City (New York), Rhode Island, Seattle/King County (Washington), Tennessee, Texas, Utah, and Wisconsin.

^¶¶^ 95% CIs were calculated after detrending underlying linear changes in weekly incidence rates using piecewise linear regression. Each CI represents the remaining variation in observed weekly incidence rates and resulting incidence rate ratios. The number of observations informing each CI reflects the number of weeks per period: October–November (7), and December (4).

*** https://www.cdc.gov/nchs/data/statnt/statnt24.pdf

Peaks in age-standardized case and death rates occurred during August (>95% of infections attributed to the Delta variant); case rates also peaked during the 2 weeks ending December 18 and 25 (39% and 72% infections attributed to the Omicron variant, respectively) (Figure). IRRs during the pre-Delta period and period of Delta predominance periods were relatively stable, followed by declines corresponding to transitions in variant prevalence (Supplementary Figure, https://stacks.cdc.gov/view/cdc/113543). Differences in case rates between fully vaccinated persons with and without a booster dose decreased over time; however, more protection was afforded from booster doses, even during Omicron emergence.

**FIGURE:**
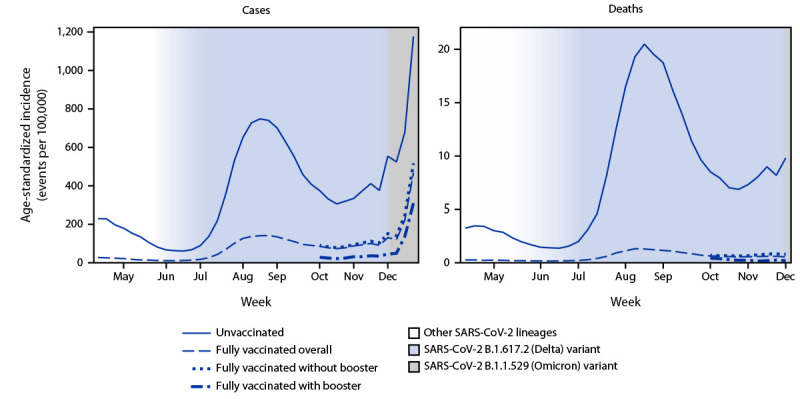
Weekly trends in age-standardized incidence of COVID-19 cases (April 4–December 25, 2021) and deaths (April 4–December 4, 2021) for unvaccinated compared with fully vaccinated persons,* overall and by receipt of booster doses^†^ and national weighted estimates of variant proportions^§^ — 25 U.S. jurisdictions^¶^ **Abbreviation**: FDA = Food and Drug Administration. * A COVID-19 case in a fully vaccinated person occurred when SARS-CoV-2 RNA or antigen was detected in a respiratory specimen collected ≥14 days after completing the primary series of a COVID-19 vaccine with FDA approval or emergency use authorization. A COVID-19 case in an unvaccinated person occurred when the person did not receive any FDA–authorized COVID-19 vaccine doses before the specimen collection date. Excluded were partially vaccinated persons who had received at least one FDA–authorized or approved vaccine dose but did not complete a primary series ≥14 days before collection of a specimen with SARS-CoV-2 RNA or antigen detected. This analysis represents the combined impact of the BNT162b2 (Pfizer-BioNTech), mRNA-1273 (Moderna), and Ad.26.COV2.S (Janssen [Johnson & Johnson]) COVID-19 vaccines, which had different clinical efficacies against confirmed infection. A COVID-19–associated death occurred in a person with a documented COVID-19 diagnosis who died, and whose report local health authorities reviewed (e.g., using vital records, public health investigation, or other data sources) to make that determination. Per national guidance, this should include persons whose death certificate lists COVID-19 disease or SARS-CoV-2 as an underlying cause of death or as a significant condition contributing to death. Rates of COVID-19 deaths by vaccination status are reported based on when the patient was tested for COVID-19, not the date they died. ^†^ A COVID-19 case in a fully vaccinated person with a booster dose occurred when a person had SARS-CoV-2 RNA or antigen detected on a respiratory specimen collected ≥14 days after receipt of at least one additional or booster dose of any COVID-19 vaccine on or after August 13, 2021. On August 13, 2021, CDC recommended an additional Pfizer-BioNTech or Moderna primary series dose for persons with moderately or severely immunocompromise. On September 24, 2021, CDC recommended a Pfizer-BioNTech booster dose for certain Pfizer-BioNTech primary series recipients, including all adults aged ≥65 years and persons aged ≥18 years in certain populations and high risk occupational and institutional settings. On October 21, 2021, CDC recommended a booster dose for adults aged ≥18 years who received the Janssen vaccine and for Pfizer-BioNTech or Moderna primary series recipients, including all adults aged ≥65 years and persons aged ≥18 years in certain populations and high risk occupational and institutional settings. On November 19, 2021, and November 29, 2021, CDC expanded recommendations for booster doses to include all adults aged ≥18 years. ^§^ National weighted estimates of the proportions of infections attributed to SARS-CoV-2 variants by week are based on whole-genome sequencing results submitted to or performed by CDC (https://covid.cdc.gov/covid-data-tracker/#variant-proportions). Other lineages prior to the Delta transition included Alpha (>50%), Gamma, Epsilon, Iota, Mu, and other lineages. ^¶^ Alabama, Arkansas, California, Colorado, District of Columbia, Florida, Georgia, Idaho, Indiana, Kansas, Louisiana, Massachusetts, Michigan, Minnesota, Nebraska, New Jersey, New Mexico, New York, New York City (New York), Rhode Island, Seattle/King County (Washington), Tennessee, Texas, Utah, and Wisconsin.

## Discussion

COVID-19 vaccines reduced risks for SARS-CoV-2 infection and COVID-19–associated death during periods of Delta variant predominance and infection risk during Omicron variant emergence. Because of reporting lags, the influence of the Omicron variant on COVID-19–associated deaths by vaccination status in December could not be evaluated. Substantial case rate increases were recorded among unvaccinated and vaccinated persons when Omicron became the predominant variant in December, resulting in decreased IRRs and declining crude VE estimates (*7*). IRRs and VE were higher among persons who were fully vaccinated and had received a booster dose than among fully vaccinated persons who had not received a booster dose for cases and deaths during the period of Delta predominance and for cases during the period of Omicron emergence in December. The added benefits of booster doses were especially prominent among persons aged 50–64 and ≥65 years.

The findings in this report are subject to at least five limitations. First, booster doses could not be distinguished from additional primary doses administered to immunocompromised persons, which could result in reduced IRRs because of lower VE in this population. Second, this ecological study lacked multivariable adjustments, and causality could not be determined. Possible differences in testing, infection-derived immunity, waning of vaccine-derived immunity, or prevention behaviors by age and vaccination status might partly explain differences in rates between groups; trends are likely affected by temporal changes in testing or reporting. Third, national variant prevalence estimates were used, but prevalence differed by jurisdiction over time. Fourth, variable data linkage completeness might have resulted in misclassifications (e.g., booster doses not being linked to primary series) that could influence IRR estimates (*5*). Finally, these data represent 62% of the overall U.S. population, and therefore might not be generalizable.

Early analysis of surveillance data provided crude signals of VE that were consistent with other studies reporting decreased VE against Omicron infection compared with Delta and increased protection from booster doses compared with a primary series of COVID-19 vaccination alone^†††^ (*10*). Ongoing analyses will help monitor the impact of the Omicron variant and other emerging variants on VE against COVID-19 cases and associated deaths; rates by vaccination status will be updated monthly on CDC’s COVID Data Tracker website (*3*). All eligible persons should stay up to date with primary series, additional, and booster doses of COVID-19 vaccine.

**Summary**

**What is already known about this topic?**

Although COVID-19 vaccine effectiveness decreased with emergence of the Delta variant and waning of vaccine-induced immunity, protection against hospitalization and death has remained high.

**What is added by this report?**

In 25 U.S. jurisdictions, decreases in case incidence rate ratios for unvaccinated versus fully vaccinated persons with and without booster vaccine doses were observed when the Omicron variant emerged in December 2021. Protection against infection and death during the Delta-predominant period against infection during Omicron emergence were higher among booster vaccine dose recipients, and especially among persons aged 50–64 and ≥65 years.

**What are the implications for public health practice?**

COVID-19 vaccination protected against SARS-CoV-2 infection, even as the Omicron variant became predominant. All eligible persons should stay up to date with COVID-19 vaccination.

## Footnotes

* These authors contributed equally to this report.

† A COVID-19 case in a fully vaccinated person occurred when SARS-CoV-2 RNA or antigen was detected in a respiratory specimen collected ≥14 days after completing the primary series of a COVID-19 vaccine with Food and Drug Administration (FDA) approval or emergency use authorization. The COVID-19 case definition, including criteria to distinguish a new case from an existing case, is per the July 2021 update to the national standardized surveillance case definition and national notification for 2019 novel coronavirus disease (COVID-19) (21-ID-01) (https://ndc.services.cdc.gov/case-definitions/coronavirus-disease-2019-2021/). Fully vaccinated persons were those with a completed primary series of 2 doses of the Pfizer-BioNTech or Moderna mRNA vaccine or a single dose of the Janssen vaccine (https://www.cdc.gov/coronavirus/2019-ncov/vaccines/stay-up-to-date.html). A COVID-19 case in an unvaccinated person occurred when the person did not receive any FDA-authorized COVID-19 vaccine doses before the specimen collection date. Cases were excluded in partially vaccinated persons who received at least one FDA-authorized or approved vaccine dose but did not complete a primary series ≥14 days before collection of a respiratory specimen with SARS-CoV-2 RNA or antigen detected. Ascertaining vaccination status for COVID-19 patients through active linkage of case surveillance and immunization information systems typically assumes that cases among persons who are unmatched to the registry are unvaccinated. This analysis represents the combined impact of the Pfizer-BioNTech, Moderna, and Janssen COVID-19 vaccines, which had different clinical efficacies against confirmed infection. Information on different FDA-authorized and approved COVID-19 vaccine products, including clinical efficacy, is available online. https://www.cdc.gov/coronavirus/2019-ncov/vaccines/different-vaccines.html

§ On August 13, 2021, CDC recommended an additional Pfizer-BioNTech or Moderna primary series dose for persons moderately or severely immunocompromised (https://www.cdc.gov/media/releases/2021/s0813-additional-mRNA-mrna-dose.html). On September 24, 2021, CDC recommended a Pfizer-BioNTech booster dose for certain Pfizer-BioNTech primary series recipients, including all adults aged ≥65 years and persons aged ≥18 years in certain populations and high risk occupational and institutional settings (https://www.cdc.gov/media/releases/2021/p0924-booster-recommendations-.html). On October 21, 2021, CDC recommended a booster dose for adults aged ≥18 years who had received the Janssen vaccine and for Pfizer-BioNTech or Moderna primary series vaccine recipients, including all adults aged ≥65 years and persons aged ≥18 years in certain populations and high risk occupational and institutional settings (https://www.cdc.gov/media/releases/2021/p1021-covid-booster.html). On November 19, 2021, and November 29, 2021, CDC expanded recommendations for booster doses to include all adults aged ≥18 years (https://www.cdc.gov/media/releases/2021/s1119-booster-shots.html,https://www.cdc.gov/media/releases/2021/s1129-booster-recommendations.html).

¶ A COVID-19 case in a fully vaccinated person with a booster dose occurred when a person had SARS-CoV-2 RNA or antigen detected on a respiratory specimen collected ≥14 days after receipt of at least 1 additional or booster dose of any COVID-19 vaccine on or after August 13, 2021 (this definition does not distinguish between vaccine recipients who are immunocompromised and are receiving an additional dose versus those who are not immunocompromised and receiving a booster dose).

** A COVID-19–associated death occurred in a person with a documented COVID-19 diagnosis who died, and whose report local health authorities reviewed (e.g., using vital records, public health investigation, or other data sources) to make that determination. Per national guidance, this group should include persons whose death certificate lists COVID-19 disease or SARS-CoV-2 as an underlying cause of death or as a significant condition contributing to death (https://cdn.ymaws.com/www.cste.org/resource/resmgr/pdfs/pdfs2/20211222_interim-guidance.pdf). Rates of COVID-19 deaths by vaccination status are reported based on when the patient was tested for COVID-19, not the date the patient died.

†† National weighted estimates of the proportions of infections attributed to SARS-CoV-2 variants by week are based on analyses of whole-genome sequencing results submitted to or performed by CDC (https://covid.cdc.gov/covid-data-tracker/#variant-proportions). Analysis periods were categorized as April 4–May 29, 2021 (pre-Delta: 0.1%–7% proportion range), May 30–July 3, 2021 (Delta emergence: 14%–69%), July 4–November 27, 2021 (Delta predominance: 81%–99%), and November 28–December 25, 2021, (Omicron emergence: 1%–72%). Other lineages in the analysis period before the Delta transition included Alpha (>50%), Gamma, Epsilon, Iota, Mu, and other lineages.

§§
https://www.census.gov/programs-surveys/popest/data/tables.2019.html

¶¶
https://www.cdc.gov/nchs/data/statnt/statnt20.pdf

*** 45 C.F.R. part 46.102(l)(2), 21 C.F.R. part 56; 42 U.S.C. Sect.241(d); 5 U.S.C.0 Sect.552a; 44 U.S.C. Sect. 3501 et seq.

††† https://www.medrxiv.org/content/10.1101/2022.01.07.22268919v1.full; https://papers.ssrn.com/sol3/papers.cfm?abstract_id=4011905
